# Activated Natural Killer Cells Withstand the Relatively Low Glucose Concentrations Found in the Bone Marrow of Multiple Myeloma Patients

**DOI:** 10.3389/fonc.2021.622896

**Published:** 2021-05-20

**Authors:** Femke A. I. Ehlers, Niken M. Mahaweni, Timo I. Olieslagers, Gerard M. J. Bos, Lotte Wieten

**Affiliations:** ^1^ Department of Transplantation Immunology, Tissue Typing Laboratory, Maastricht University Medical Center+, Maastricht, Netherlands; ^2^ Department of Internal Medicine, Division of Hematology, Maastricht University Medical Center+, Maastricht, Netherlands; ^3^ GROW School for Oncology and Developmental Biology, Maastricht University, Maastricht, Netherlands

**Keywords:** NK cells, multiple myeloma, immunotherapy, tumor microenvironment, glucose

## Abstract

Infusion of *ex vivo* expanded and cytokine-activated natural killer (NK) cells is a promising alternative way to treat multiple myeloma (MM). However, the tumor microenvironment (TME) may suppress their function. While reduced glucose availability is a TME hallmark of many solid tumors, glucose levels within the TME of hematological malignancies residing in the bone marrow (BM) remain unknown. Here, we measured glucose levels in the BM of MM patients and tested the effect of different glucose levels on NK cells. BM glucose levels were measured using a biochemical analyzer. Compared to the normal range of blood glucose, BM glucose levels were lower in 6 of 9 patients (479-1231 mg/L; mean=731.8 mg/L). The effect of different glucose levels on NK cell cytotoxicity was tested in 4-hour cytotoxicity assays with tumor cells. 500 mg/L glucose (representing low range of MM BM) during the 4-hour cytotoxicity assay did not negatively affect cytotoxicity of activated NK cells, while higher glucose concentrations (4000 mg/L) diminished NK cell cytotoxicity. Since clinical application of NK cell therapy might require *ex vivo* expansion, expanded NK cells were exposed to a range of glucose concentrations from 500-4000 mg/L for a longer period (4 days). This did not reduce cytotoxicity or IFN-γ secretion nor affected their phenotypic profile. In summary, low glucose concentrations, as found in BM of MM patients, by itself did not compromise the anti-tumor potential of IL-2 activated NK cells *in vitro*. Although follow up studies in models with a more complex TME would be relevant, our data suggest that highly activated NK cells could be used to target tumors with a reduced glucose environment.

## Introduction

In the last decade, considerable effort has been put in the development of NK cell-based immunotherapy to treat cancer patients due to the clinical potential and good safety profile of NK cells. Multiple clinical trials using either autologous or allogeneic NK cells in various types of hematological- and solid cancers have demonstrated that NK cells could exert anti-tumor responses in patients without significant toxicity (1–3). Mostly, *ex vivo* expanded and cytokine-activated NK cells are used to create highly cytotoxic NK cells. Nonetheless, despite these initially hopeful clinical outcomes, the therapeutic efficacy of NK cell-based immunotherapy could be improved by increasing NK-cell numbers, enhancing NK-cell activation, improving NK-cell tumor-targeting capacity, and improving *in vivo* NK-cell persistence (1).

The expansion and persistence of NK cells *in vivo* has been demonstrated to be positively correlated with the clearance of leukemic cells in patients receiving adoptive NK cell therapy (4). However, the microenvironment of tumor cells could be unfavorable and even suppressive for NK cells allowing tumor cells to escape the NK-cell antitumor response. To be able to survive in the tumor microenvironment (TME), NK cells require cytokines such as IL-2 or IL-15 that can be produced by several cell types present in the TME, but the available amount might not be enough (5, 6). The presence of other cytokines such as transforming growth factor-beta (TGF-ß) and IL-10 in the TME may play a role in the suppression of IL-2 production (7). NK cell antitumor capacity can be hindered by TME factors such as myeloid derived suppressor cells (8), hypoxia (9, 10), or factors released by the tumor cells such as prostaglandin E2, TGF-ß, IL-10, reactive oxygen species, and arginase (11–13). Additionally, the metabolic microenvironment of tumor cells could inhibit the antitumor response of immune cells such as cytotoxic T cells and NK cells (14). To sustain their growth and survival, tumor cells frequently undergo metabolic reprogramming, allowing the enhancement of glucose uptake and metabolism. This process takes place not only within a hypoxic region but also in the area where sufficient oxygen is available, a phenomenon known as aerobic glycolysis or “the Warburg effect” (15). Aerobic glycolysis is favorable for proliferating cells since it can provide both bioenergetics and biosynthesis requirements better than oxidative phosphorylation (OxPhos) (14). Due to high rates of glycolysis, the glucose supply in the tumor microenvironment can be limited. In solid tumors, glucose availability is inversely correlated with the distance from the capillaries to the tumor and glucose levels as low as 20 mg/L have been reported for colon cancer with great intratumoral variability (16, 17). To our knowledge, there is not much known about glucose levels within the microenvironment of hematological cancers.

Aerobic glycolysis appeared to be not only advantageous for tumor cells. Immune cells, such as cytotoxic T cells, have been shown to require a switch to aerobic glycolysis to exert their effector function (18). Since both tumor cells and T cells are glycolytic, metabolic competition can occur within the tumor microenvironment. The glycolytic activity of the tumor cells can cause depletion of extracellular glucose thereby limiting the availability of glucose to T cells (19). In mice, it has been demonstrated that this metabolic competition hindered T-cell metabolism resulting in a defective IFN-γ production which is crucial for antitumor response (20, 21). In NK cells, aerobic glycolysis has also been shown to be important for a potent NK-cell effector function. In mice, resting NK cells preferred OxPhos for their metabolism while highly activated NK cells enhanced especially glycolysis and to a lower extend OxPhos (22–24). In humans, NK cells upregulated both glycolysis and OxPhos upon cytokine stimulation with IL-2 or IL-12/15 (25). Additionally, CD56 bright NK cells were found to be metabolically more active than CD56 dim NK cells (25). The same group also showed that elevated levels of OxPhos were essential for NK cell effector cytotoxicity and IFN-γ production.

Our group focuses on the development and refinement of NK-cell based immunotherapy to treat patients with cancer, especially multiple myeloma (MM) as there is no cure available to date for MM. We envision to inject a high number of highly activated NK cells to patients with MM. Glucose levels in the MM microenvironment remain unknown but may be important in controlling the anti-MM response of NK cells as NK cells use glucose as primary source of energy (26). We, therefore, aimed to explore the possible consequences of MM metabolic microenvironment on the antitumor potential of activated and expanded NK cells. First, we investigated the glucose levels present in the microenvironment of patients having active MM to define the relevant *in vitro* experimental conditions. Second, based on these results, we performed 4-hour cytotoxicity assays *in vitro* to study the effect of short-term exposure to different glucose concentrations on NK cell cytotoxicity against tumor cells. Third, we studied the influence of longer exposure to the different glucose concentrations on expanded NK cells to evaluate whether NK cell effector functions could be optimized by adapting glucose levels during expansion. The results from this current study give us a better understanding whether *in vivo* glucose concentrations should be a concern for the NK-cell based immunotherapy and whether eventually an intervention might be needed to improve the therapy.

## Materials and Methods

### Glucose Measurement

Leftover fresh BM samples were obtained from MM patients with active disease. The use of leftover material from clinical procedures did not require ethical approval in the Netherlands under the Dutch Code for Proper Secondary Use of Human Tissue. None of the patients objected to the use of leftover material. When feasible, samples were measured directly as a whole BM harvest. Otherwise, samples were centrifuged with speed 1170 g for 15 minutes at 4° Celsius, followed by harvesting of the “plasma” fraction which was stored in -20° Celsius before the glucose measurement was performed using YSI biochemical analyzer (Salm en Kipp, BV). Freezing the samples had minor influence on the glucose concentration, similar to variation between duplicates. Samples were measured in duplicate and the average per sample was reported.

### Cell Lines and Culture Media

The K562 cell line, purchased from ATCC, was cultured in IMDM (Gibco) and supplemented with 10% fetal calf serum (FCS) (Greiner Bio-One International, GmbH), 100 U/mL penicillin (Gibco), and 100 µg/mL streptomycin (Gibco) (1% Pen/Strep). The RPMI-8226 cell line, purchased from DSMZ, was cultured in standard RPMI-1640 medium. Standard RPMI-1640 medium refers to RPMI-1640 medium containing 2000 mg/L glucose (Cat. 11554516, ThermoFisher) and was supplemented with 10% FCS and 1% Pen/Strep for all cultures. Glucose-free RPMI-1640 medium (Cat. 11560406, ThermoFisher) was supplemented with D-(+)-Glucose (Sigma) as indicated in the individual figures and with 10% FCS and 1% Pen/Strep. All cell culture experiments were performed at 37° C in an incubator with 5% CO_2_ and 21% O_2_ (Sanyo MCO-20AIC, Sanyo Electric Co, Japan).

### NK Cell Culture and NK Cell Expansion

NK cells were isolated from anonymous buffy coats (Sanquin blood bank, Maastricht). The use of buffy coats does not need ethical approval in the Netherlands under the Dutch Code for Proper Secondary Use of Human Tissue. Peripheral blood mononuclear cells were isolated from the buffy coats by density centrifugation using Lymphoprep (Axis-Shield). Subsequently, NK cells were isolated using the NK cell isolation kit according to the manufacturer’s protocol (Miltenyi Biotec, GmbH). For experiments in **Figure 2**, NK cells were activated overnight with 1000 U/mL recombinant human IL-2 (Proleukin, Novartis) either in standard RPMI-1640 medium containing 2000 mg/L glucose (Gibco, **Figure 2A**) or in glucose-free RPMI-1640 medium (Gibco) supplemented with the glucose concentration indicated in the **Figure 2B** (named ‘culture condition’). For experiments in **Figures 3** and **4**, NK cells were expanded from CD3-depleted PBMCs in the presence of 1000 U/mL IL-2 in alpha-medium (Biochrom, GmbH) supplemented with 10% human serum (Milan Analytica, AG), 2mM L-GlutaMax (Gibco), 1.3 g/L sodium bicarbonate (Biochrom), 2000 mg/L glucose (Sigma), and 0.5% Gentamycin-Sulphate (Gibco). The cells were expanded with IL-2 for 16-22 days during which the NK cells expanded 10- to 20-fold. After expansion, the NK cells were subsequently cultured for 4 days in glucose-free RPMI-1640 medium supplemented with the glucose concentration indicated in the figure and with 1000 U/mL IL-2 (culture condition), followed by cytotoxicity assays and ELISA assays.

### Labeling of Tumor Cells Lines for Cytotoxicity Assays

One day prior to the cytotoxicity assay, 2 x 10^6^ cells/ml K562 cells or RPMI-8226 cells were labeled with 3 µl Vybrant CM-DiI Cell-Labeling solution (Thermo Fisher) in PBS according to the manufacturer’s instruction. After adding CM-DiI to the cell suspension, cells were incubated for 5 minutes at 37°C followed by 15 minutes at 4°C in the fridge. After the last incubation, cells were washed 2 times with PBS and centrifuged (280g for 8 minutes at room temperature). K562 or RPMI-8226 cells were then resuspended in IMDM or standard RPMI-1640 medium, respectively, supplemented with 10% FCS and 1% Penicillin/Streptomycin and incubated overnight at 37°C.

### Cytotoxicity Assay

On the day of the assay, the DiI-labeled tumor cells were harvested and 2x10^4^ cells per well were plated in a 96-wells plate for the cytotoxicity assay. NK cells were harvested, washed and co-cultured with the tumor cells in 1:1 Effector : Target (E:T) ratio for 4 hours in the presence of different glucose concentrations (named ‘killing condition’) as indicated in the figures. Specific cytotoxicity was calculated as follows: (% dead tumor cells - % spontaneous tumor cell death)/(100% - % spontaneous tumor cell death) x100.

### Staining and Flow Cytometry

After a 4-hour cytotoxicity assay, cells were washed with PBS (Gibco) and stained for dead cells using Live/Dead^®^ Fixable Aqua Dead Cell Stain Kit (Molecular Probes™) for 30 minutes on ice in the dark. Cells were further washed with PBS buffer (PBS, 1% FCS) and fixed with 1% paraformaldehyde in PBS solution. For NK cell phenotyping, expanded NK cells were harvested after culturing in either 500, 2000, or 4000 mg/L glucose for 4 days. Subsequently, NK cells were washed in PBS and first stained for dead cells using Live/Dead^®^ Fixable Aqua Dead Cell Stain Kit (Molecular Probes™) for 30 minutes on ice in the dark, and subsequently stained for the following surface markers for 30 minutes on ice and in the dark: CD3-APC-Vio770, -VioBlue or -PerCP-Vio700; CD56-PE-Vio770 or -APC-Vio770; KIR2DL2/3-PE; KIR3DL1-PerCP; NKG2A-APC; KIR2DL1-FITC; NKp30-PE-Vio770; NKp44-VioBright; NKp46-PE; NKG2D-APC; PD1-VioBright; DNAM1-PE; CD96-PE-Vio770; NKG2C-APC; LAG3-VioBlue; TIM3-APC; TIGIT-PE. FMO controls were stained for Live/Dead, CD3 and CD56. All antibodies were purchased from Miltenyi Biotech. All flow cytometric analyses were performed with BD FACS Canto II. Data were analyzed with FlowJo 10.1r5 64-bit software.

### ELISA Assay

Supernatants of the cytotoxicity assays were collected and analyzed for IFN-γ levels using the PeliKine compact™ ELISA kit (Sanquin). The samples were thawed at room temperature and diluted 1:2 before use. ELISA assays were performed according to the manufacturer’s instructions.

### Statistical Analysis

All statistical analysis was performed with GraphPad Prism 8.3 (GraphPad Software Inc, San Diego, CA, USA) using non-parametric paired t-test (Wilcoxon matched pairs test) for figure 2A or 2-way ANOVA when comparing multiple groups. * indicates a *p-value* of <0.05, ** indicates a *p-value* of <0.01.

## Results

### Glucose Concentrations in the BM of MM Patients Are on Average Lower Than Normal Blood Glucose Levels

**Figure 1:**
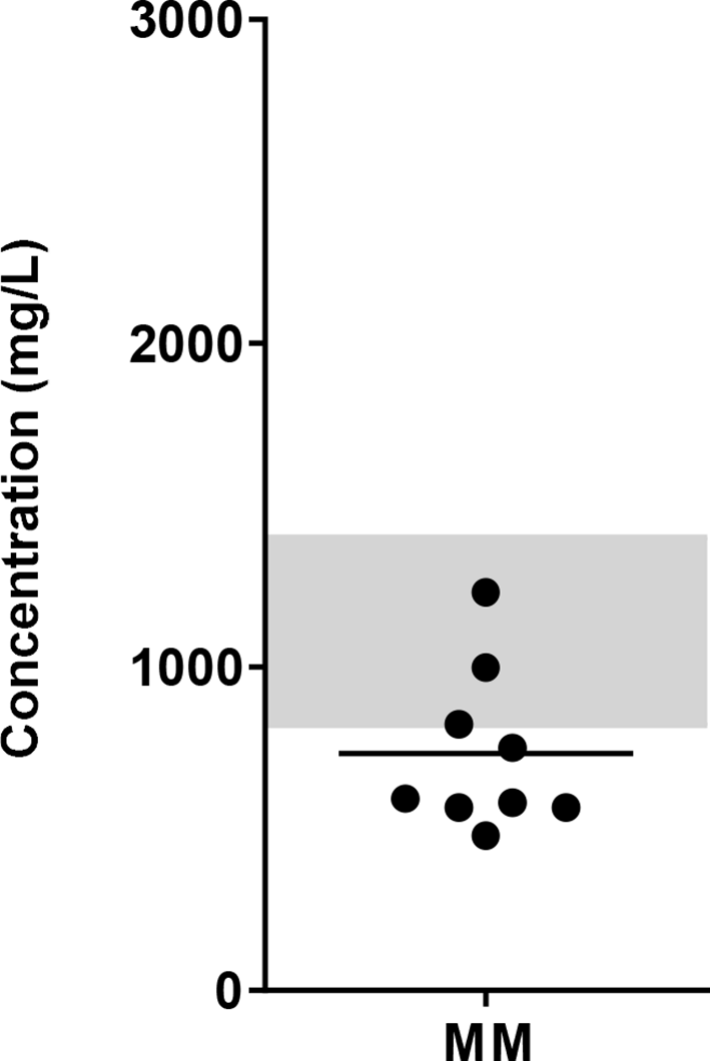
Glucose concentration in the BM of MM patients. BM samples from newly diagnosed MM patients were collected and glucose concentrations were determined using biochemical analyzer (YSI). Grey bar indicates the reference value range for normal fasting blood glucose (4.4 – 6.1 mmol/L or 820 – 1100 mg/L up to 1400 mg/L postprandial). n = 9 subjects.

To get an indication of glucose levels in the bone marrow (BM), glucose levels were measured in BM samples from MM patients. The glucose concentration in the BM of MM patients ranged between 479 to 1231 mg/L (mean = 731.8 mg/L, SD = 247.6). Compared to normal glucose levels in peripheral blood, which are < 1100 mg/L (<6.1 mmol/L) fasting or <1400 mg/L (<7.8 mmol/L) random (27), 6 of 9 MM patients had lower glucose levels with the lowest concentration reported here being twice as low as normal blood glucose levels (**Figure 1**).

### Relatively Low Glucose Concentrations Present During Killing Do Not Affect NK Cell Killing Capacity While High Glucose Reduced Killing Efficacy

**Figure 2:**
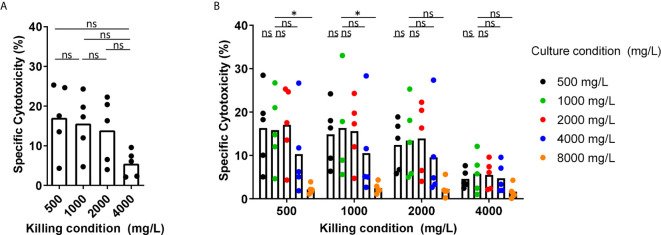
Short-term exposure to lower glucose concentrations does not reduce NK cell cytotoxicity while higher glucose levels reduce NK cell cytotoxicity. **(A)** NK cells were overnight activated with IL-2 in standard RPMI-1640 medium with 2000 mg/L glucose, followed by a 4h cytotoxicity assay with K562 cells at 1:1 E:T ratio in different glucose concentrations (killing condition). **(B)** NK cells were cultured in different glucose concentrations overnight (culture condition) in presence of IL-2, followed by a 4h cytotoxicity assay with K562 cells at 1:1 E:T ratio in different glucose concentrations (killing condition). Tumor cells killed by NK cells are denoted as percentage specific cytotoxicity. Bars show the average of n=5 donors in 3 independent experiments. Each dot represents the average of a technical duplicate. *p < 0.05. ns, not significant.

Glucose has been described to be an important fuel for NK cells. Therefore, we tested the effect of the glucose levels as observed in BM of MM patients, the glucose levels in normal blood, and the glucose levels used in culture media on the cytotoxic capacity of NK cells derived from healthy donors. We activated NK cells overnight with 1000 U/mL IL-2 in standard RPMI-1640 medium containing 2000 mg/L glucose and then used the NK cells in a 4-hour cytotoxicity assay against K562 cells at 500, 1000, 2000, or 4000 mg/L glucose. In the presence of 1000 mg/L, which is representative for the glucose concentration in blood, NK cells killed 19% of K562 cells on average. The presence of 500 mg/L glucose (representing the low range of MM BM) during the cytotoxicity assay did not negatively affect the killing capacity of IL-2 activated NK cells as compared to conditions with 1000 mg/L, representing blood glucose, or 2000 mg/L, which is present in standard culture media (**Figure 2A**). Higher glucose concentration of 4000 mg/L, as used in high glucose culture media, reduced the cytotoxicity of NK cells to 5.5% on average as compared to 500 mg/L, 1000 mg/L and 2000 mg/L glucose (p= 0.06) (**Figure 2A**).

Because NK cells could be exposed for a longer period to the BM glucose levels while traveling through the BM, we investigated whether overnight exposure to different glucose concentrations affected NK cell cytotoxicity. The exposure to 500 mg/L up to 4000 mg/L glucose during overnight incubation (named ‘culture condition’) did not result in a lower cytotoxicity against K562 cells regardless of the glucose concentrations present during the killing process (named ‘killing condition’) (**Figure 2B**). After observing a reduction in NK cell-mediated killing with 4000 mg/L glucose in **Figure 2A**, we included an even higher glucose concentration of 8000 mg/L for overnight culture. In the presence of this extremely high glucose, NK cell cytotoxicity was reduced to less than 5% in all killing conditions (**Figure 2B**). This reduction in NK cell cytotoxicity was unlikely due to high osmolarity caused by the high glucose levels as we did not see a reduction in NK cell cytotoxicity when NK cells were cultured in the presence of 1000 mg/L glucose and 7000 mg/L Mannitol (**Supplementary Figure S1**).

In summary, these results showed that the presence of 500 mg/L glucose, representative for the rather low glucose concentration in BM of MM patients, during the process of killing or during overnight incubation did not reduce the NK cell tumor-killing capacity in our *in vitro* setting. Quite the opposite, the presence of higher levels glucose concentration during killing or overnight activation (4000 and 8000 mg/L) diminished NK cell cytotoxic capacity.

### Expanded NK Cells Exposed to Relatively Low Glucose Levels Remain Effective Against Tumor Cells

Clinical application of NK cells might require *ex vivo* NK cell expansion to reach the large numbers of NK cells needed for NK cell infusions. As this expansion may also metabolically change the cells, we investigated how the killing capacity of expanded NK cells was affected by short-term exposure (4 hours, killing condition) or long-term exposure (4 days, culture condition) to glucose levels ranging between 500 mg/L and 4000 mg/L.

**Figure 3:**
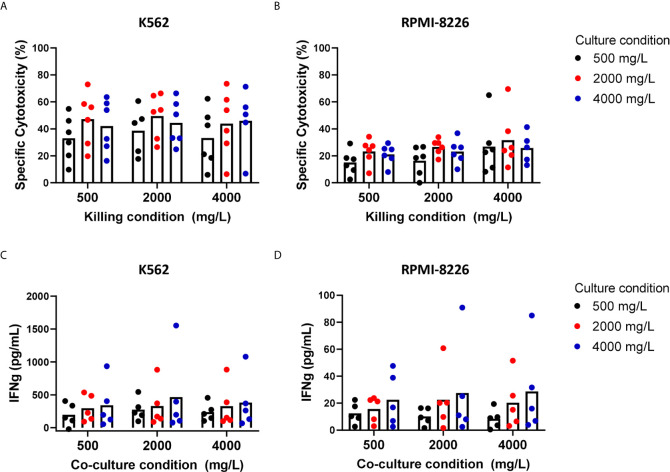
Short-term and long-term exposure to low or high glucose levels do not reduce effector functions of expanded NK cells. NK cells were expanded with 2000 mg/L glucose and subsequently cultured in different glucose concentrations for 4 days (culture condition) followed by a 4h cytotoxicity assay (killing condition) with K562 cells or RPMI8226 cells at 1:1 E:T ratio in different glucose concentrations. Tumor cells killed by NK cells are denoted as percentage specific cytotoxicity **(A, B)**. After the co-culture with tumor cells, supernatants were collected and analyzed for secretion levels of IFNγ by ELISA **(C, D)**. Bars show the average of n=5-6 donors in individual experiments. For none of the conditions a p-value <0.05 was observed.

With 2000 mg/L glucose present during the cytotoxicity assay, expanded NK cells killed on average 49.5% K562 cells. RPMI-8226 cells, a MM cell line, were more resistant to NK cell-mediated killing than K562 cells and 26.6% RPMI-8226 cells were killed on average at 2000 mg/L glucose. Compared to this condition with 2000 mg/L glucose, the average cytotoxic potential of expanded NK cells against K562 and RPMI-8226 cells was not altered when NK cells were exposed to 500 mg/L or 4000 mg/L glucose during the killing process (**Figures 3A, B**). This result indicated that short exposure to varying glucose concentration did not influence the cytotoxic potential of expanded NK cells and differed from the freshly isolated NK cells that seemed to have reduced cytotoxicity with 4000 mg/L glucose during killing (**Figure 2**).

Next, we examined the effect of a four-day exposure to the different glucose concentrations on NK cell cytotoxicity. Compared to the culture condition of 2000 mg/L glucose, the average cytotoxicity of both K562 and RPMI-8226 was around 10% lower when NK cells were cultured in 500 mg/L glucose, however this did not reach significance. The average cytotoxicity of NK cells cultured in 4000 mg/L was not different from the culture condition with 2000 mg/L (**Figures 3A, B**). The NK cell viability remained the same in all conditions when NK cells were cultured in 500, 2000, or 4000 mg/L for 4 days (**Supplementary Figure S2A**). Despite some donor variation, expanded NK cells of all donors maintained their cytotoxic capacity largely independent of the glucose concentration present during 4-day culture.

Besides production of cytotoxic granules, NK cells are known for their secretion of inflammatory cytokines such as IFN-γ and TNFα. NK cell-derived IFN-γ has multiple functions including support of antigen presentation and induction of a Th1 response, which is important for polarizing an adaptive immune response against tumor cells (28). To investigate if the secretion of IFN-γ was influenced by the different glucose levels, the supernatants of NK- and tumor cell co-cultures were analyzed for IFN-γ secretion by ELISA. Cultured in the normal culture condition with 2000 mg/L glucose, expanded NK cells secreted on average 329 pg/mL IFN-γ with K562 cells as target cells and 23 pg/mL with RPMI-8226 as target cells, showing that K562 induced a much more potent IFN-γ response in NK cells than RPMI-8226 cells (**Figures 3C, D**). Without target cells, expanded NK cells did not secrete IFN-γ (**Supplementary Figure S2B**). The average level of IFN-γ production was comparable for all the tested glucose levels during the short-term co-culture with tumor cells.

Compared to the 4-day culture condition with 2000 mg/L glucose, the average IFN-γ secretion remained constant when the expanded NK cells were exposed to 500 mg/L or 4000 mg/L for 4 days. This was found for both target cell lines K562 and RPMI-8226. These data showed that a potent IFN-γ response, comparable to the amount secreted with 2000 mg/L glucose, was still observed independent of the glucose concentration during short- or long-term culture.

**Figure 4:**
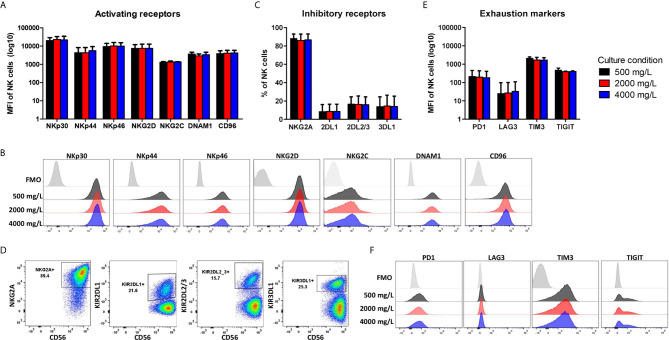
Phenotype profile of expanded NK cells is not altered by long-term exposure to low or high glucose levels. NK cells were expanded with 2000 mg/L glucose and subsequently cultured in different glucose concentrations for 4 days (culture condition). NK cells were stained with antibodies against activating receptors **(A, B)**, inhibitory receptors **(C, D)** and exhaustion markers **(E, F)** and analyzed by flow cytometry. Bar graphs **(A, C, E)** depict the average expression of n=2 donors for NKG2C, TIM3, TIGIT and n=5 donors for all other markers, error bars indicate SD. FMO values were subtracted from MFI expression values. Representative histograms **(B, F)** or dot plots **(D)** of one donor are shown with the FMO of one of the three glucose concentrations as FMOs overlapped tightly (**Supplementary Figure S3**).

During *ex vivo* expansion, NK cells frequently alter their phenotype resulting in high NKG2A expression and low expression of killer-cell immunoglobulin-like receptors (KIRs). To test if the NK cell phenotype changed by culturing in low or high glucose during the 4-day culture period, NK cells were stained for several surface markers. The gating strategy is shown in **Supplementary Figure S3**. The activating receptors NKp30, NKp46, DNAM1 and NKG2C are constitutively expressed on NK cells with NKG2C being expressed on a subset of NK cells. NKp44 and CD96 expression are induced upon activation (29). After expansion of NK cells in the presence of IL-2, the NK cells had a highly activated phenotype and expressed all seven activating receptors (**Figures 4A, B**). With 86%, the majority of expanded NK cells was NKG2A positive and on average 10-18% of the NK cells expressed one or multiple KIR receptors (**Figures 4C, D**). Moreover, extensive cytokine activation of NK cells can lead to expression of exhaustion markers. The NK cells expressed TIM3 and TIGIT but only low levels of PD1 and LAG3 after expansion with IL-2 (**Figures 4E, F**). Expression of all investigated surface molecules was irrespective of the glucose concentrations present during culture, resulting in NK cells with a rather activating phenotypic profile.

In summary, the effector functions and the phenotypic profile of expanded NK cells were not influenced by variation in glucose levels during the killing process or during culture. Follow up studies that take multiple other TME factors and nutrient state into account, could be useful to further determine the impact of glucose on NK cells.

## Discussion

To date, not much is known about glucose concentrations in the BM. As glucose is the primary fuel for NK cells, we aimed to gain more insight on the effect of glucose levels in the TME of MM on overnight cytokine-activated or *ex vivo* expanded NK cells and their antitumor response. First, we showed that glucose concentrations in the BM of MM patients with active disease were, in most cases, lower than normal blood glucose levels. Since we included only 9 patients in our study, our observation should be confirmed in a larger cohort. With this new insight on glucose concentrations in BM, we tested the effect of different glucose levels on freshly isolated- or expanded NK cells *in vitro*. As lowest glucose concentration we chose 500 mg/L since this represents the lower limit of BM glucose concentrations in MM patients. We observed that short-term exposure to 500 mg/L glucose did not have a detrimental effect on the killing capacity of overnight-activated NK cells. This may be because glucose was not completely depleted. 500 mg/L glucose is half of the normal concentration found in blood, where NK cells are known to be potent killers. Moreover, the majority of NK cells in the assays were CD56 dim NK cells. Both human NK cell subsets are functionally different and have been shown to possess different metabolic requirements as well: CD56 bright NK cells, which are the main cytokine producers have been shown to have a higher rate of glucose uptake and they appear to be metabolically more active than CD56 dim NK cells upon cytokine stimulation (25). Therefore, CD56 bright peripheral blood NK cells may be more likely to suffer more from the restricted glucose in the environment than the CD56 dim subset. In addition, during activation, NK cells are able to perform metabolic reprogramming and upregulate both glycolysis and OxPhos (30). Since we used high dose IL-2 activated NK cells, these cells could have become more or less independent on the availability of glucose. Unlike T cells that are more dependent on glucose availability to become activated, NK cells might be the better tumor cell killers in the area where low glucose concentrations are located. Moreover, to fully conclude whether this would also be true for solid tumors, the glucose levels should be reduced even further as glucose levels in solid tumors can be much lower than 500 mg/L (17). In addition, it would be interesting to further evaluate whether our findings are also true in a situation where not only glucose is reduced, but also other nutrients are limited.

Interestingly, high glucose concentrations of 4000 mg/L and even more obvious with 8000 mg/L glucose did result in a reduction of NK cell cytotoxicity in our study. This observation was highly unlikely due to high molarity since we did not observe the effect with mannitol. Our finding was in line with a previous study on unactivated human NK cells showing that short-term exposure of NK cells to 8000 mg/L glucose led to inhibition of NK cell cytotoxicity, probably due to an increase in intracellular calcium ion concentration to such high levels that it inhibited cytotoxic efficiency (31, 32).

Many clinical protocols, aiming at NK cell infusion as a mean for cancer immunotherapy, will require infusion of extremely high numbers of NK cells, which necessitates *ex vivo* NK cell expansion. We therefore anticipated that this expansion period could provide an opportunity to either prime NK cells for the metabolic conditions in the tumor or enhance their function or persistence by interfering with their metabolic programming. For these experiments, we again chose 500 mg/L glucose as low, 2000 mg/L as normal, and 4000 mg/L as high glucose concentration, representing the *in vivo* BM concentrations in MM patients, glucose concentrations in standard-, and in high glucose culture media, respectively. Our data with IL-2 expanded NK cells implied that a period of acclimatization to a higher glucose concentration did not result in an altered NK cell cytotoxic capacity. Additionally, a period of acclimatization to a lower glucose level for 4 days after expansion did not reduce NK cell-mediated killing. For murine tumor-infiltrating T cells, it has been shown that inhibition of glycolysis during the *ex vivo* expansion could prime T cells towards enhanced persistence and overall anti-tumor response upon transfer into tumor-bearing immunodeficient mice (33). In human NK cells expanded with membrane-bound IL-21 K562 feeder cells, highly functional licensed NK cells used both glycolysis and OxPhos and had a greater glycolytic capacity than less functional, unlicensed NK cells that relied on OxPhos alone (34). Inhibition of NK cell cytotoxicity in highly functional NK cells was only observed after vigorous glucose deprivation and the use of metabolic inhibitors overnight (34). This provides the functional NK cells with a greater flexibility to generate energy and indicates that the cytotoxic functions can be independent of the available glucose levels. We also found that the IFN-γ response of the expanded NK cells during a 4h cytotoxicity assay was largely unaffected by the glucose concentrations that we tested. A previous study using murine NK cells showed that inhibition of glycolysis reduced IFN-γ production by NK cells when triggered by engagement of activating receptors while cytokine (IL-12/IL-18)-induced IFN-γ production remained unaffected by inhibition of glycolysis (23). Similar to murine NK cells, human cytokine-activated NK cells continued to produce IFN-γ when exposed to short-term glucose deprivation of 4 hours (35) or long-term low glucose levels (as low as 0.01 mM) for two days (36). However, CD56bright NK cells showed a defective IFN-γ production when the glycolysis rate was limited for a period of 18 hours (25) underlining the difference between CD56dim and CD56bright NK cells.

In our study, we observed a rather small reduction in glucose availability in the BM of MM patients as compared to normal peripheral blood values but we did not determine availability of other nutrients in the MM patients. In our *in vitro* assays, we reduced the amount of glucose, while other nutrients could contribute to NK cell efficacy as well. Glucose has been described as the key metabolic fuel for NK cells [summarized in (37)], but further research would be required to determine if a small reduction in glucose availability might have more impact in combination with other nutrient deficiencies, such as glutamine, or other TME factors that may suppress NK cell effector function or alter NK cell metabolism. While NK cells do not use glutamine as fuel for OxPhos, glutamine deficiency can impair NK cell functions due to the loss of the transcription factor cMYC (26). Alternatively, the combination with TGF-β, frequently present in the MM TME (38), may enhance the effect of limited glucose as TGF-β has been shown to reduce the NK cells level of OxPhos and glycolysis (39). Hypoxia is another TME factor that can limit NK cell functions. We have previously shown that IL-2 activated NK cells retain their cytotoxic capacity when exposed to hypoxia (9). However, a shift towards glycolysis is expected when oxygen is low and it is therefore important to study the effects of varying glucose concentrations on activated NK cells in models resembling a complex TME including factors like hypoxia and altered levels of nutrients other than glucose.

Even though we show that activated and expanded NK cells can cope with a low glucose environment, combination therapy should be considered to achieve better NK cell efficacy in MM patients. We have previously described several strategies how the NK cell potency could be enhanced e.g. by the combination with monoclonal antibodies such as Daratumumab (40). Other options could include targeting of the TME to create a less NK cell suppressive TME. In MM, inhibitors of mechanistic target of rapamycin (mTOR) are tested to target the metabolism of MM cells and its TME cells that overexpress mTOR (41, 42). Dual inhibitors targeting mTORC1/mTORC2 could potentially be an interesting drug to slow tumor cell growth in combination with NK cell transfer to kill tumor cells. However, mTOR inhibitors can also suppress immune cells (22, 43) and combination therapy approaches should be tested in carefully designed studies as timing of the drugs and NK cell infusion may be very important. Alternatively, it would be relevant to explore in further studies whether manipulating NK cell metabolism during *ex vivo* expansion could be used to potentiate NK cell effector function in the MM TME.

In conclusion, our current findings showed that exposure to a relatively low glucose concentration, as found in the BM of MM patients, for either short-term or long-term culture did not have a detrimental effect on the NK cell cytotoxic capacity against tumor cells in our *in vitro* setup. Our data suggests that IL-2 activated and expanded NK cells could be well suited to function in a tumor environment where glucose availability is limited. Although this is positive news for NK-cell based immunotherapy, future studies are needed to investigate if these observations also hold true for the *in vivo* situation in patients with MM or other cancers.

## Data Availability Statement

The raw data supporting the conclusions of this article will be made available by the authors, without undue reservation.

## Ethics Statement

Ethical review and approval was not required for the study on human participants in accordance with the local legislation and institutional requirements. Written informed consent for participation was not required for this study in accordance with the national legislation and the institutional requirements.

## Author Contributions

Conceptualization, NM and FE. Methodology, NM, FE, and TO. Formal analysis, NM and FE. Investigation, NM, FE, and TO. Resources, NM, GB. Data curation, NM and FE. Writing—original draft preparation, NM and FE. Writing, review and editing, TO, LW, and GB. Supervision, LW and GB. Project administration, NM and FE. Funding acquisition, LW and GB. All authors contributed to the article and approved the submitted version.

## Funding

This study was funded by a Grant from Kankeronderzoeksfonds Limburg (KOFL).

## Conflict of Interest

GB is Chief Executive Officer/Chief Medical Officer/Co-founder of CiMaas, BV, Maastricht, The Netherlands. CiMaas is producing an *ex vivo* expanded NK cell product that will be used to treat myeloma patients.

The remaining authors declare that the research was conducted in the absence of any commercial or financial relationships that could be construed as a potential conflict of interest.
