# Supplementary material for: Activated Natural Killer Cells Withstand the Relatively Low Glucose Concentrations Found in the Bone Marrow of Multiple Myeloma Patients
Source: Front Oncol. 2021 May 20;11:622896. doi: 10.3389/fonc.2021.622896 (PMC8174784; doi:10.3389/fonc.2021.622896)
Supplement: Supplementary file 1 [file DataSheet_1.pdf]

Supplementary Material

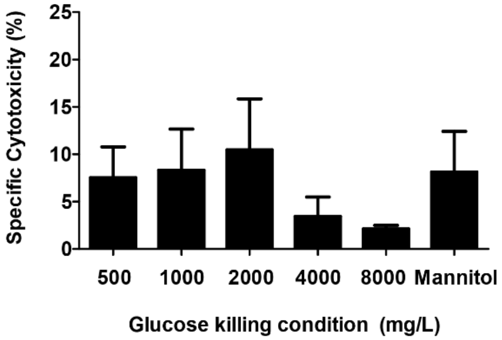

Supplementary Figure S1. High osmolarity is not the cause of a reduced cytotoxic capacity of NK cells due to high glucose concentration during culture. NK cells stimulated with 1000 U/ml IL-2 were cultured overnight in the presence of different glucose concentrations or high concentration of Mannitol equivalent to 8000 mg/L glucose. The following day, NK cells were co-cultured with K562 cells in 1:1 E:T ratio in a 4-hour cytotoxicity assay in different glucose concentrations as indicated. Dead cells were stained with Live/Dead Marker. Percentage of tumor cells killed by NK cells are denoted as percentage specific cytotoxicity. n = 2 donors in independent experiments.

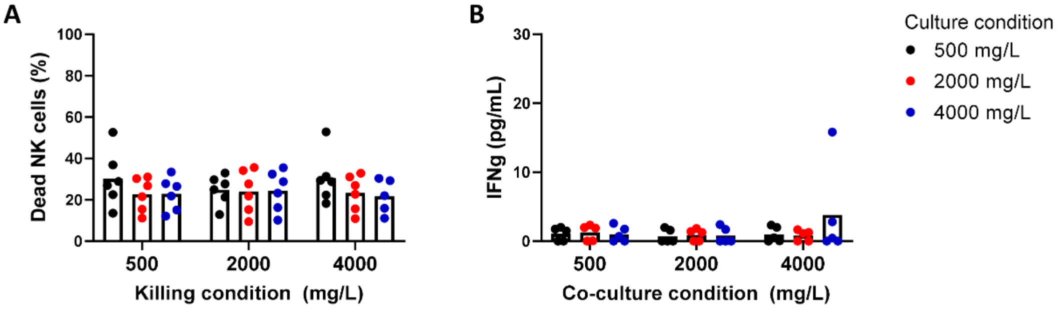

Supplementary Figure S2. Effect of short- or long-term exposure to low or high glucose on NK cells. NK cells were expanded with 2000 mg/L glucose and subsequently cultured in different glucose concentrations for 4 days (culture condition). **(A)** NK cell viability was assessed by staining with a Live/Dead marker and analysis with flow cytometry. **(B)** IFN-γ secretion of expanded NK cells without presence of target cells, assessed by ELISA. Bars show average of n=5-6 donors in individual experiments.

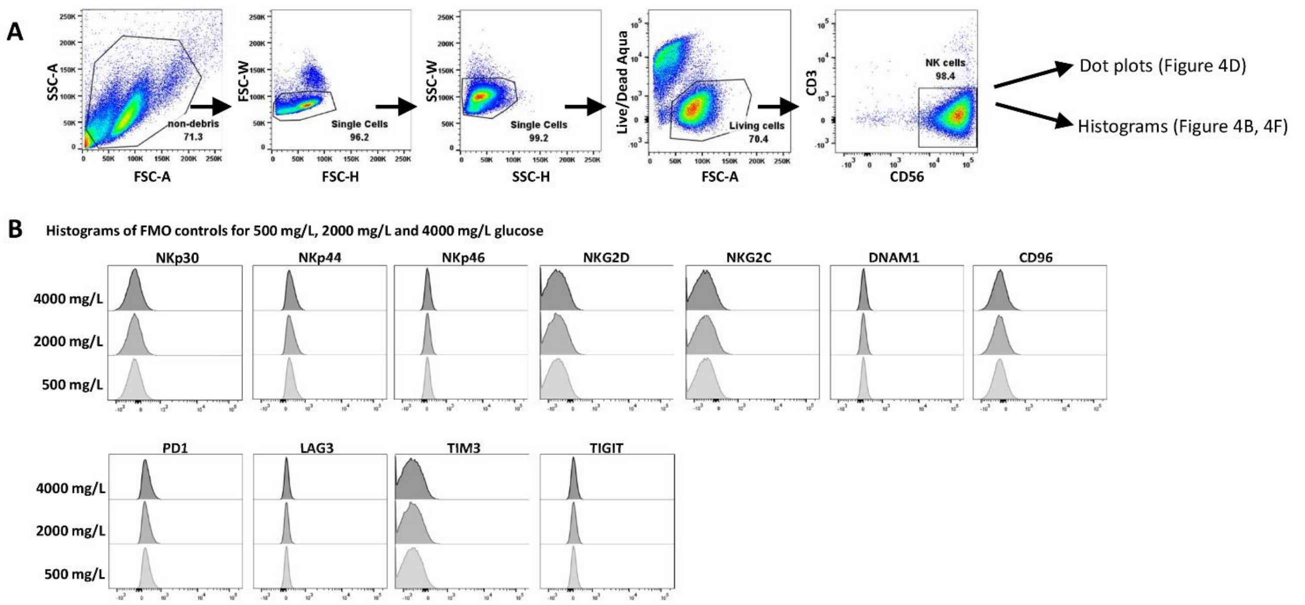

Supplementary Figure S3: Analysis of the phenotypic profile of expanded NK cells. (A) Gating strategy was as follows: Debris was gated out and single living cells were selected before gating on CD3- CD56+ NK cells, followed by gating on the inhibitory receptors NKG2A, KIR2DL1, KIR2DL2/3, KIR3DL1 that present as subpopulations of NK cells (see Figure 4D) and activating receptors and exhaustion markers are presented as histograms (see Figure 4B and 4F). **(B)** Histograms of FMO controls for all glucose culture conditions (500 mg/L, 2000 mg/L, 4000 mg/L) are shown of a representative donor.
